# Neuroimaging Findings of Psychosis in Alzheimer's Disease: A Systematic Review

**DOI:** 10.1002/brb3.70205

**Published:** 2024-12-31

**Authors:** Fardin Nabizadeh, Shadi Sheykhlou, Sara Mahmoodi, Elham Khalili, Rasa Zafari, Helia Hosseini

**Affiliations:** ^1^ School of Medicine Iran University of Medical Sciences Tehran Iran; ^2^ Alzheimer's Disease Institute Tehran Iran; ^3^ Medical Laboratory Department Iran University of Medical Sciences Tehran Iran; ^4^ Universal Scientific Education and Research Network (USERN) Bandar Abbas Hormozgan Iran; ^5^ Cardiovascular Research Center Hormozgan University of Medical Sciences Bandar Abbas Iran; ^6^ School of Medicine Tehran University of Medical Science Tehran Iran; ^7^ Faculty of Medicine Tehran University of Medical Sciences Tehran Iran

**Keywords:** Alzheimer's disease, delusion, hallucination, neuroimaging, psychosis

## Abstract

**Background:**

Previous studies on neuroimaging findings in Alzheimer's disease (AD) patients with hallucinations and delusions have yielded inconsistent results. We aimed to systematically review neuroimaging findings of delusions and hallucinations in AD patients to describe the most prominent neuroimaging features.

**Methods:**

We performed a comprehensive search in three online databases, including PubMed, Scopus, and Web of Science in June 2023. We included studies that reported neuroimaging features of AD patients with delusion, hallucination, or psychosis.

**Results:**

After the screening, 34 studies with 2241 AD patients were eligible to be included in our qualitative synthesis. On the basis of the included studies, there are significant changes in the volume and perfusion levels of broad brain areas, including the hippocampus, amygdala, insula, cingulate, occipital, frontal, prefrontal, orbitofrontal, temporal, and parietal cortices in these patients. Moreover, AD patients with psychosis, hallucinations, or delusions reflected different EEG waves compared to AD patients without these disorders.

**Conclusion:**

The results of our review provided evidence about the neuroimaging alterations in AD patients suffering from psychosis, hallucinations, and delusions using different imaging methods. AD patients with psychosis, hallucinations, or delusions have significant differences in the volume and perfusion levels of various brain regions along with alterations in EEG waves and biological molecules compared to patients with only AD.

## Graphical abstract

Neuroimaging alterations in Alzheimer's disease patients with delusion, hallucination, or psychosis.

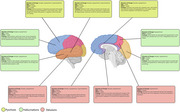

## 1 Introduction

Neuropsychiatric symptoms, also referred to as behavioral and psychological symptoms of dementia, are highly prevalent, affecting approximately 97% of individuals along the dementia continuum (Steinberg et al. 2008). Among these neuropsychological symptoms (NPS), psychotic symptoms, characterized by the presence of delusions and/or hallucinations, are a hereditary trait found in 40%–60% of individuals with Alzheimer's disease (AD) (Sweet et al. 2003). A systematic review revealed that among AD patients with psychosis, 23% displayed delusions exclusively, 5% had hallucinations alone, and 13% experienced both delusions and hallucinations (Sweet et al. 2003). Those with AD and psychotic symptoms typically experience more severe cognitive impairment and executive dysfunction and are prone to neuropsychiatric disturbances such as aggregation, agitation, and depression (Fischer et al. 2012; Zahodne et al. 2015). This results in a reduced quality of life, accelerated disease progression, higher mortality rates, increased economic burden, and heightened stress for their caregivers (Lanctôt et al. 2017; Scarmeas et al. 2005; Wilson et al. 2006). Recently, Cummings et al. (2020) revised diagnostic criteria for psychosis in individuals with major and mild neurocognitive disorders, addressing a critical need for clearer guidelines to aid clinical practice, research, and treatment development. The revised criteria incorporate feedback from global experts and expand upon previous definitions to improve the accuracy and applicability of diagnoses.

Research on neuroimaging findings in AD patients with psychosis has yielded somewhat inconsistent results. Additionally, the majority of previous studies have primarily focused on delusions rather than hallucinations or the combined presence of delusion and hallucination symptoms. In terms of structural and functional magnetic resonance imaging (MRI) modalities evaluating volumetric measurements, AD patients experiencing delusions have shown increased atrophy in frontotemporal and hippocampal regions (Ismail et al. 2012; Qian, Schweizer, et al. 2019; Lee et al. 2019). Specifically, assessments of individuals with delusional misidentification syndromes have revealed parahippocampal atrophy (Förstl et al. 1994; Yang et al. 2023). Gray matter atrophy in the cerebellum and parietal lobe, without frontal lobe involvement, has also been reported. However, a study using data from the Alzheimer's Disease Neuroimaging Initiative (ADNI) has suggested a potential link between posterior cortical atrophy, frontal circuits, and the default mode network (DMN) and delusional development in AD patients (Fischer et al. 2016). On the other hand, another study using the ADNI data observed supramarginal atrophy of the parietal lobe when exclusively studying AD patients with hallucinations (Donovan et al. 2014; Wang, Shang, et al. 2022). Furthermore, investigating those AD patients who exhibited both delusion and hallucination, there was no evidence of any alteration in functional connectivity within the DMN (Balthazar et al. 2014; Cheng, Huang, et al. 2024). Concerning imaging modalities measuring regional blood flow and glucose metabolism, such as single‐photon emission computed tomography (SPECT) and fluorine‐18 fluorodeoxyglucose positron emission tomography (^18^F‐FDG‐PET), the majority of studies have consistently identified hypometabolism and hypoperfusion patterns in the right frontal and temporal cortices (Ismail et al. 2012; Murray, Kumar, et al. 2014). However, it is important to note that the majority of these studies have predominantly focused on delusions. More recent investigations using ^18^F‐FDG‐PET and SPECT have demonstrated specific findings, including hypometabolism in the orbitofrontal region and hypoperfusion in the right hemisphere consisting of inferior temporal gyrus, parahippocampal cortex, posterior insula, and amygdala (Murray, Kumar, et al. 2014; Blanc et al. 2014; Nakatsuka et al. 2013). Bilateral hypoperfusion in the temporal poles has also been reported in these investigations (Nakatsuka et al. 2013). Furthermore, in the context of nuclear imaging, research focusing on AD patients with psychosis has been restricted and often constrained by small sample sizes. Nonetheless, in one study, no link was found between the 5‐hydroxytryptamine 2A receptor (5‐HT2A) and psychosis symptoms (Santhosh et al. 2009). In contrast, another investigation exploring dopamine receptors revealed an elevated number of striatal D2/3 receptors in AD patients who were experiencing psychosis (Reeves et al. 2009). Chronic psychosis‐related stress can induce neuroinflammation, contributing to AD progression by promoting amyloid‐beta (Aβ) plaque and tau tangle formation (Bisht et al. 2018; Chen et al. 2023). Conversely, AD‐related neurodegeneration, particularly in the frontal and temporal lobes, can lead to impaired cognitive control, manifesting as psychosis (Ismail et al. 2022). Additionally, shared genetic factors and vascular dysfunctions might predispose individuals to both conditions, further linking psychosis with AD onset and progression (Govindpani et al. 2019; Cheng, Li, et al. 2024).

Given the lack of definitive findings and recognizing the importance of studying both individual and combined aspects of delusions and hallucinations, our primary aim was to systematically review neuroimaging findings of delusions and hallucinations in AD patients to describe the most prominent neuroimaging features. Our results may help clinicians to better diagnose as well as predict future clinical symptoms of AD patients with hallucination, delusion, or psychosis.

## 2 Methods and Materials

The present study was conducted following the Preferred Reporting Items for Systematic Reviews and Meta‐Analyses (PRISMA) statement (Liberati et al. 2009).

### 2.1 Search Strategy

We performed a comprehensive search in three online databases including PubMed, Scopus, and Web of Science using the following terms in June 2023: “Alzheimer, psychosis, hallucination, Charles Bonnet syndrome, delusion, positron emission tomography, PET, beta‐amyloid, amyloid, amyloid‐β, amyloid deposition, PiB, Pittsburgh, florbetapir, flortaucipir, tau, tau deposition, FDG, fluorodeoxyglucose, DTI, diffusion tens, imaging, microstructure, anisotropy, Diffusivity, functional magnetic resonance imaging, functional MRI, fMRI, rsfMRI, resting‐state fMRI, Brain mapping, Structural MRI, voxel‐based morphometry, gray matter, white matter, VBM, MRI, magnetic resonance imaging, atrophy, hippocampus, EEG, electroencephalography, electroencephalogram.” Additional studies were identified via a manual search of reference lists. Full search strategy is available in Supporting Information 1.

### 2.2 Inclusion Criteria

We included studies that reported neuroimaging features of AD patients with delusion, hallucination, or psychosis with a minimum sample size of five. We excluded reviews, case reports, letters, and non‐English studies.

### 2.3 Study Selection

Two independent researchers (S.S., S.M.) screened the title and abstract in the first step. Then, the same researchers reviewed the full text of the remaining studies to identify eligible studies. Any disagreements were resolved by consulting with the third reviewer (F.N.).

### 2.4 Data Extraction

Two investigators extracted the required data based on a predesigned sheet independently. The following data were extracted: author, year of publication, study design, sample size, age, gender, AD diagnosis criteria, MMSE score, type of psychosis symptoms, number of patients with psychosis symptoms, and findings.

### 2.5 Quality Assessment

We used the Newcastle‐Ottawa scale (NOS) to assess the quality of the included studies with the highest possible score of 8 (Lo et al. 2014).

## 3 Results

### 3.1 Study Characteristics

**FIGURE 1:**
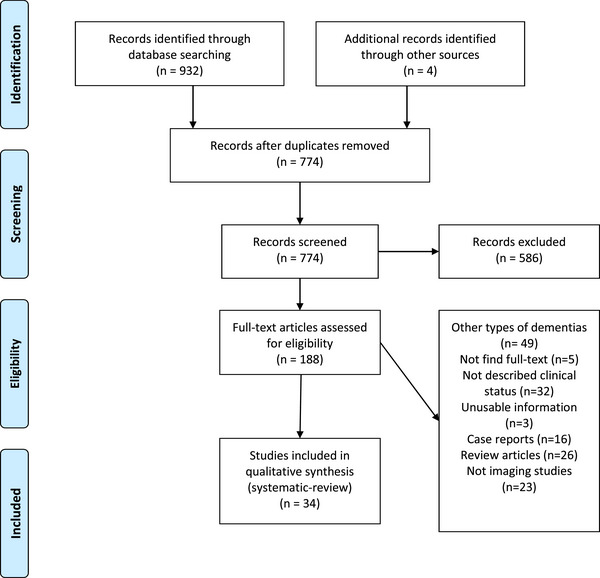
PRISMA flow diagram depicting the flow of information through the different phases of a systematic review.

A total of 774 studies were identified via database search and manual addition after duplicate removal (Figure 1). After title and abstract screening, 586 studies were excluded, and the remaining studies underwent careful review. Finally, 34 studies were eligible to be included in our qualitative synthesis (Qian, Schweizer, et al. 2019; Lee et al. 2019, 2022; Nakatsuka et al. 2013; Bruen et al. 2008; D'Antonio et al. 2019; Dauwan et al. 2018; Fan et al. 2023; Förstl et al. 1993; Fukuhara et al. 2001; Geroldi et al. 2002; Gomar et al. 2022; Hirono et al. 1998; Holroyd et al. 2000; Howanitz et al. 1995; Kotrla et al. 1995; Lin et al. 2006; Makovac et al. 2016; Manca et al. 2023; Matsuoka et al. 2010; McLachlan et al. 2018; Mega et al. 2000; Moran et al. 2008; Nakaaki et al. 2013; Nakano et al. 2006; Nomura et al. 2012; Palmqvist et al. 2011; Qian, Fischer, et al. 2019; Staff et al. 1999, 2000; Sultzer et al. 2003; Sultzer et al. 2014; Sweet et al. 2002; Whitehead et al. 2012).

Among the included studies, 22 were cross‐sectional, and 12 were longitudinal (Table 1). The total number of AD patients was 2241 with mean age ranging between 60 and 82. The quality of included studies assessed by NOS was acceptable with a mean score of 7.08 (Supporting Information 2).

**TABLE 1:** Demographical and clinical characteristics of included studies.

Author	Year	Country	Study design	Total number of AD patients	Age	Disease duration	Number of females	MMSE score	AD confirmation criteria	Total number of AD patients with psychosis (hallucination and delusion)	Type of psychosis	Neurobehavioural assessment	Comparing groups	AD biomarkers	Imaging method
Fan	2023	China	Cross‐Sectional	80	77.19 ± 9.052	7.86 ± 5.405	63	10.84 ± 7.156	NIA‐AA	36	Delusion	NPI	AD patients without delusions		MRI
Manca	2023	United Kingdom	Longitudinal	126	75.67 ± 6.16	NR	52	25.10 ± 3.72	NIA‐AA	63	Delusion	NPI	AD patients without delusions		MRI
Gomar	2022	USA	Cross‐sectional	67	79.23 ± 6.71	NR	27	20.94 (5.01)	NIA‐AA	17	Psychotic (delusions and hallucinations)	NPI‐Q	AD patients without psychosis	Amyloid positive	PET
Lee	2021	South Korea	Longitudinal	109	71.57 § 7.42	NR	65	NR	NINCDS/ADRDA	27	Incident psychosis	Psychosis of Alzheimer's disease and related dementias	AD patients without psychosis		MRI
Qian	2019	Canada	Longitudinal	30	76.9 ± 7.0	NR	19	NR	NIA‐AA	15	Delusion	NPI	AD patients without delusions		fMRI
D'Antonio	2019	Italy	Longitudinal	42	72.9 ± 7	5.6 ± 2.1	12	20.2 ± 4.2	NIA‐AA	17	Psychotic (delusions and hallucinations)	NPI	AD patients without psychosis		MRI
Lee	2018	South Korea	Longitudinal	74	60	2.07 ± 2.36	17	17.34 ± 4.10	NINCDS/ADRDA	26	Psychotic (delusions and hallucinations)	Psychosis of Alzheimer's disease and related dementias	AD patients without psychosis		MRI
Qian	2018	Canada	Longitudinal	59	78.0 ± .1	NR	46	18.09 ± 6.8	NR	23	Delusion	NPI‐Q	AD patients who did not develop delusions		MRI
Dauwan	2018	Netherlands	Longitudinal	173	69.41	3	19	24	NINCDS/ADRDA	36	Hallucination	NPI	AD patients without hallucinations		EEG
McLachlan	2017	United Kingdom	Longitudinal	104	73.4 ± 5.8	3.4 ± 2.5	70	21.3 ± 4.8	DSM‐IV	47	Psychotic (delusions and hallucinations)	NPI	AD patients without psychosis		MRI
Makovac	2015	Italy	Cross‐sectional	58	71.9 ± 7.2	NR	37	19.0 ± 3.9	NINCDS/ADRDA	16	Psychotic (delusions and hallucinations)	NPI	AD patients without behavioral disorders and psychological symptoms		DTI and MRI
Nakatsuka	2013	Japan	Cross‐sectional	59	81.9 ± 5.7	NR	NR	15.2 ± 4.1	NINCDS/ADRDA	27	Delusion	BEHAVE‐AD‐FW	AD patients without delusions		SPECT
L. Sultzer	2013	USA	Cross‐sectional	88	78 ± 8.0	3.2 ± 2.4	17	16.0 ± 5.0	NINCDS/ADRDA	28	Delusion	NPI	AD patients without delusions		FDG‐PET
Nomura	2012	Japan	Cross‐sectional	87	75.7 ± 6.8	NR	65	17.4 ± 5.3	NINCDS/ADRDA	87	Delusion of persecution, delusion of theft, delusional jealousy, PBS, belief that one's spouse or others are not who they claim to be, belief that his/her house is not his/her home, delusion of abandonment, and delusion relating to the television	NPI	None		SPECT
Nakaaki	2012	Japan	Longitudinal study	53	78.2 ± 5.7	1.1 ± 0.8	39	21.1 ± 3.6	NINCDS/ADRDA	35	Delusion	NPI	AD patients without delusions		MRI
Palmqvist	2011	Sweden	Longitudinal study	259	75 ± 6.4	NR	176	21.4 ± 5	NINCDS/ADRDA	74	Delusions and hallucinations	NPI	NONE		MRI‐CT
Whitehead	2011	United Kingdom	Cross‐sectional	113	75.7 ± 5.6	3.6 ± 2.1	74	19.3 ± 4.6	DSM‐IV and NINCDS/ADRDA	23	Paranoid delusions	NPI	AD patients without delusions		MRI
Matsuoka	2010	Japan	Cross‐sectional	35	76.5 ± 6.0	2.9±2.8	30	20.8 ± 3.8	NINCDS/ADRDA	14	Delusion	NPI	AD patients without delusions		SPECT
Buren	2008	United Kingdom	Cross‐sectional	31	77.1	NR	12	23.30 ± 2.8	NINCDS‐ADRDA	6	Psychotic (delusions and hallucinations)	NPI	AD patients without psychosis		MRI
Moran	2007	USA	Cross‐sectional	103	79 ± 6.0	NR	68	16.5 ± 6.2	NINCDS/ADRDA	51	Psychotic (delusions and hallucinations)	BEHAVE‐AD	AD patients without psychosis		SPECT
Lin	2006	Taiwan	Cross‐sectional	10	73 ± 6	NR	4	16 ± 9	NR	5	Visual hallucination	History of Visual hallucination	AD patients without Visual hallucination		MRI
Nakano	2006	Japan	Cross‐sectional	64	74.7	NR	NR	16.8 ± 5.9	NINCDS/ADRDA	25	Delusion	NPI	AD patients without delusions		SPECT
L. Sultzer	2003	USA	Cross‐sectional	25	69.5 ± 6.7	4.2 ± 1.9	1	16.5 ± 6.0	NINCDS/ADRDA	19	Delusion	Neurobehavioral rating scale (NRS)	NONE		FDG‐PET
Geroldi	2002	Italy	Cross‐sectional	41	76 ± 8	NR	17	22 ± 3	NINCDS‐ADRDA	19	Delusions	NPI	AD patients without delusions		CT
Sweet	2001	USA	Cross‐sectional	28	77.2 ± 8.0	11.0 ± 3.2	18	NR	DSM III‐R	12	Psychotic (delusions and hallucinations)	Neurobehavioral rating scale (NRS)	AD patients without psychosis		MRS
Fukahara	2001	Japan	Cross‐sectional	18	76	NR	NR	20 ± 2.9	NINCDS‐ADRDA	9	Delusion of theft	NPI	AD patients without delusions		SPECT
Mega	2000	USA	Cross‐sectional	20	NR	NR	19	14.30 ± 2.31	NINCDS/ADRDA	10	Hallucinations: visual, auditory	NPI	NONE		SPECT
Staff	2000	United Kingdom	Cross‐sectional	45	NR	NR	32	18.3 ± 3.4	NINCDS/ADRDA	20	Autobiographic delusions	NR	AD patients without delusions		SPECT
Holroyd	2000	USA	Cross‐sectional	14	74.2 ± 3.1	NR	10	19.8 ± 5.2	NINCDS‐ADRDA	7	Visual hallucinations	History of visual hallucination	AD patients without hallucinations		MRI
Staff	1999	United Kingdom	Cross‐sectional	33	70.7 ± 6.9		24	19.11 ± 6.37	NINCDS/ADRDA	18	Delusion	NR	AD patients without delusions		SPET imaging
Hirono	1998	Japan	Cross‐sectional	65	71.2 ± 6.9	32.7 ± 18.5	48	18.5 ± 4.2	DSM‐IV and ANOVA	26	Delusions	NPI	AD patients without delusions		PET and MRI
Kotrla	1995	USA	Longitudinal study	46	77.5 ± 9.6	NR	NR	Baseline visit (Npre): 24.9 ± 4.7	DSM‐III‐R,	30	Psychotic (delusions and hallucinations)	DSM‐III‐R	AD patients without delusions and hallucination		SPECT
Howanitz	1995	USA	Cross‐sectional	32	78	4.36	21	NR	DSM‐III‐R and CERAD	20	Psychotic (delusions and hallucinations)	NR	AD patients without psychosis		MRI
Forstl	1994	Germany	Longitudinal	50	81.3 ± 6.2	7.7 ± 4.6	43	5.3	NINCDS‐ADRDA	31	Psychotic (delusions and hallucinations)		AD patients without psychosis		EEG and CT

Abbreviations: AD, Alzheimer's disease; CT, computed tomography.; DSM, Diagnostic and Statistical Manual of Mental Disorders; DTI, diffusion tensor imaging; EEG, electroencephalogram; fMRI, magnetic resonance imaging; MRI, magnetic resonance imaging; NIA‐AA, National Institute on Aging and Alzheimer's Association; NINCDS/ADRDA, National Institute of Neurological and Communicative Disorders and Stroke and the Alzheimer's Disease and Related Disorders Association; NR, not reported; PET, positron emission tomography; SPECT, single‐photon emission computerized tomography.

## 4 MRI

### 4.1 AD and Psychosis

AD with psychosis is initially linked to the reduced size of the right hippocampus irrespective of the frontal region size (Lee et al. 2019). A longitudinal study among 109 patients with AD showed a reduction in the cortical volume or thickness of the medial temporal lobe (MTL) in AD patients with psychosis (Table 2) (Lee et al. 2022). The other longitudinal study on 42 AD patients showed the same results. AD patients with psychosis exhibited greater atrophy in the right interior–inferior temporal lope (fusiform gyrus) as well as a greater rate of atrophy in the right insula compared with nonpsychotic AD patients (D'Antonio et al. 2019). AD confirmation in the first study was based on NINCDS/ADRDA, and in the second one, it was based on NIA‐AA. A cross‐sectional study using structural MRI and diffusion tensor imaging (DTI) in 2015 on 58 patients with AD (mean age: 71, female: 63.7%, MMSE score: 19, AD confirmation: NINCDS/ADRDA) demonstrated various connections between microstructural damage of white matter in 4 divisions of the corpus callosum and the atrophy of gray matter in AD patients with psychotic symptoms (Makovac et al. 2016).

**TABLE 2:** Neuroimaging findings of included studies.

Author	Year	Total number of AD patients	Total number of AD patients with psychosis (hallucination and delusion)	Type of psychosis	Comparing groups	Imaging method	Findings
MRI							
Howanitz	1995	32	20	Psychotic (delusions and hallucinations)	AD patients without psychosis	MRI	There was correlation between presence of hallucinations and overall atrophy, right and left lateral ventricle size. There was no correlation for delusions
Holroyd	2000	14	7	Visual hallucinations	AD patients without hallucinations	MRI	AD patients with visual hallucinations had a significantly smaller occipital/whole brain ratio than AD patients without visual hallucinations
Lin	2006	10	5	Visual hallucination	AD patients without visual hallucination	MRI	AD patients with visual hallucinations demonstrated a higher occipital periventricular hyperintensities score compared to those without visual hallucinations. However, the occipital deep white matter hyperintensities score was uniformly zero in both patient groups
Buren	2008	31	6	Psychotic (delusions and hallucinations)	AD patients without psychosis	MRI	Significant correlations were observed between high delusion scores and decreased gray matter density values in the right inferior frontal gyrus and inferior parietal lobule. Furthermore, in the left hemisphere, significant correlations were found in the inferior and medial frontal gyri and in the claustrum, indicating associations between delusion severity and reduced gray matter density in these regions
Whitehead	2011	113	23	Paranoid delusions	AD patients without delusions	MRI	In female participants exhibiting paranoid delusions, there was an observed decrease in cortical thickness within the left medial orbitofrontal and left superior temporal regions, regardless of cognitive decline. However, male participants with delusions did not exhibit any notable differences compared to males without delusions
Nakaaki	2012	53	35	Delusion	AD patients without delusions	MRI	The presence of structural deviations in the frontal and medial temporal lobes may exhibit a potential association with the subsequent emergence of delusional symptoms among AD individuals
McLachlan	2017	104	47	Psychotic (delusions and hallucinations)	AD patients without psychosis	MRI	A significant overall impact of different subtypes of psychosis was observed in the region of interest associated with the ventral visual stream. This effect was primarily attributed to a reduction in volume of the left parahippocampal gyrus. Subsequent comparisons among the various psychosis subtypes revealed that the reduction in left parahippocampal volume remained significant, and it was particularly pronounced in individuals with the misidentification and mixed subtypes compared to those in the paranoid and nonpsychotic groups
Lee	2018	74	26	Psychotic (delusions and hallucinations)	AD patients without psychosis	MRI	The reduction in size of the right hippocampus is linked to the initiation of AD with psychosis irrespective of the size of the frontal region
Qian	2018	59	23	Delusion	AD patients who did not develop delusions	MRI	The AD developed delusions consistently demonstrated reduced volume of gray matter in the frontal region, whereas both groups exhibited a decline in gray matter within frontotemporal brain regions over time
Dauwan	2018	173	36	Hallucination	AD patients without hallucinations	EEG	AD with hallucination showed lower peak frequency, a2‐ and b‐power, and a2‐functional connectivity, but higher d‐power compared to AD without hallucination
Qian	2019	30	15	Delusion	AD patients without delusions	fMRI	The AD patients with delusional symptoms demonstrated a significant decrease in connectivity between the left inferior parietal lobule (IPL) and the remaining regions within the default mode network (DMN)
D'Antonio	2019	42	17	Psychotic (delusions and hallucinations)	AD patients without psychosis	MRI	AD patients with psychosis exhibited greater atrophy in the right interior‐inferior temporal lope, including the fusiform gyrus as well as greater rate of atrophy in the right insula than nonpsychotic patients
Lee	2021	109	27	Incident psychosis	AD patients without psychosis	MRI	The study findings indicated that a reduction in the cortical thickness or volume of specific subregions within the medial temporal lobe is associated with an elevated risk of developing psychosis in patients with AD
Fan	2023	80	36	Delusion	AD patients without delusions	MRI	AD patients with delusion had higher WMH volume in left occipital lobe
Palmqvist	2011	259	74	Delusions and hallucinations	NONE	MRI‐CT	Lacunes in the left basal ganglia were significantly associated with delusions and hallucinations
Makovac	2015	58	16	Psychotic (delusions and hallucinations)	AD patients without behavioral disorders and psychological symptoms	DTI and MRI	Different patterns of correlation between microstructural white matter damage of four parts of the corpus callosum and gray matter atrophy in AD patients exhibiting psychotic symptoms
Manca	2023	126	63	Delusion	AD patients without delusions	MRI	AD patients who subsequently developed delusions had greater GM volume loss in both the left and the right caudate nuclei. Moreover, greater longitudinal GM loss in delusional patients was also observed in the bilateral medio‐temporal ROIs (bilateral parahippocampal gyri and left hippocampus), in the right anterior cingulate cortex and in posterior hubs of the DMN (bilateral precuneus and left posterior cingulate cortex)
SPECT							
Kotrla	1995	46	30	Psychotic (delusions and hallucinations)	AD patients without delusions and hallucination	SPECT	Patients presenting with delusions exhibited decreased perfusion in the left frontal lobe relative to the right frontal lobe. Conversely, patients experiencing hallucinations demonstrated decreased perfusion in the parietal lobe
Staff	1999	33	18	Delusion	AD patients without delusions	SPET	In individuals with AD, delusions may potentially correlate with regions of reduced blood flow in the right anterior hemisphere. However, the presence of delusions does not necessarily indicate a distinct focal site of hypoperfusion in these patients
Mega	2000	20	10	Hallucinations: visual, auditory	NONE	SPECT	Patients diagnosed with AD who exhibit symptoms of psychosis may demonstrate an imbalanced impairment in the frontal lobes as well as associated subcortical and parietal structures
Staff	2000	45	20	Autobiographic delusions	AD patients without delusions	SPECT	The group of AD autobiographic delusions showed a noteworthy region of reduced blood flow in the right frontal lobe in comparison to the other two groups. This region of reduced blood flow encompassed specific portions of Brodmann's areas 9 and 10. Previous research has highlighted the involvement of region 9 in facilitating the retrieval of episodic memories
Fukahara	2001	18	9	Delusion of theft	AD patients without delusions	SPECT	AD patients with delusion of theft showed significant hypoperfusion in the right media posterior parietal region compared to patients without delusions
Nakano	2006	64	25	Delusion	AD patients without delusions	SPECT	The AD patients with delusions exhibited a significant reduction in perfusion levels within multiple brain regions of the right hemisphere, including the prefrontal cortex, anterior cingulate gyri, inferior to middle temporal cortices, and parietal cortex
Moran	2007	103	51	Psychotic (delusions and hallucinations)	AD patients without psychosis	SPECT	Our study revealed that female AD patients exhibiting psychotic symptoms had decreased perfusion levels in the right inferolateral prefrontal cortex and inferior temporal regions, when compared to female patients without such symptoms. Conversely, male AD patients with psychotic symptoms exhibited higher perfusion levels in the right striatum, in contrast to male patients without such symptoms
Matsuoka	2010	35	14	Delusion	AD patients without delusions	SPECT	When comparing AD patients with delusions to a healthy control group, a notable reduction in regional cerebral blood flow was observed in the right anterior insula of patients with delusions
Nomura	2012	87	87	Delusion of persecution, delusion of theft, delusional jealousy, PBS, belief that one's spouse or others are not who they claim to be, belief that his/her house is not his/her home, delusion of abandonment, and delusion relating to the television	None	SPECT	Delusion beliefs about home, phantom boarder symptom, abandonment, and misidentification were correlated with perfusion changes in the right temporal pole, medial frontal, and precentral regions. Delusion regarding television and persecution, with corresponding perfusion changes in the precuneus, insula, and thalamus. Delusions of abandonment and jealousy, related to perfusion changes in the right inferior temporal and frontal regions, middle frontal gyrus, insula, and posterior cingulate gyrus
Nakatsuka	2013	59	27	Delusion	AD patients without delusions	SPECT	In the AD with delusion, there was a significant decrease in cerebral blood flow observed in the bilateral temporal lobes, with a dominance toward the right hemisphere. Specifically, reduced CBF was found in the right inferior temporal gyrus and right amygdala, when compared to the AD without delusion
PET							
Hirono	1998	65	26	Delusions	AD patients without delusions	PET and MRI	In AD patients with delusions, there was a significant increase in glucose metabolism observed in the left inferior temporal gyrus, whereas there was a reduction in glucose metabolism in the left medial occipital region when compared to those without delusions
L. Sultzer	2003	25	19	Delusion	NONE	FDG‐PET	Hypometabolism in two distinct regions of the right prefrontal cortex, including the superior dorsolateral area (specifically the lateral aspect of Brodmann's area 8) and the inferior frontal pole (Brodmann's area 10), is positively correlated with the manifestation of delusional ideation among AD patients
L. Sultzer	2013	88	28	Delusion	AD patients without delusions	FDG‐PET	Delusions observed in AD are characterized by dysfunction within specific regions of the frontal and temporal cortices. Patients presenting delusional symptoms exhibited decreased metabolic activity in the right lateral frontal cortex, orbitofrontal cortex, and bilateral temporal cortex
Gomar	2022	67	17	Psychotic (delusions and hallucinations)	AD patients without psychosis	PET	Psychosis is associated with increases in tau pathology in frontal, medial temporal, and occipital cortices
Other							
Forstl	1994	50	31	Psychotic (delusions and hallucinations)	AD patients without psychosis	EEG and CT	Patients with hallucinations had slightly higher ventricle‐brain ratios and higher mean theta and delta powers
Sweet	2001	28	12	Psychotic (delusions and hallucinations)	AD patients without psychosis	MRS	Subjects with AD exhibiting psychosis symptoms showed significant increases in glycerophosphoethanolamine levels and significant decreases in *N*‐acetyl‐l‐aspartate. These findings suggest that the excessive deterioration of neocortical neuronal and synaptic integrity may serve as the underlying structural foundation for psychosis in individuals with AD
Geroldi	2002	41	19	Delusions	AD patients without delusions	CT	AD with delusions showed temporal horns larger to the right and the frontal horn to the left
Dauwan	2018	173	36	Hallucination	AD patients without hallucinations	EEG	AD with hallucination showed lower peak frequency, a2‐ and b‐power, and a2‐functional connectivity, but higher d‐power compared to AD without hallucination

Abbreviations: AD, Alzheimer's disease; CT, computed tomography.; DSM, Diagnostic and Statistical Manual of Mental Disorders; DTI, diffusion tensor imaging; EEG, electroencephalogram; fMRI, magnetic resonance imaging; MRI, magnetic resonance imaging; NIA‐AA, National Institute on Aging and Alzheimer's Association; NINCDS/ADRDA, National Institute of Neurological and Communicative Disorders and Stroke and the Alzheimer's Disease and Related Disorders Association; NR, not reported; PET, positron emission tomography; SPECT, single‐photon emission computerized tomography.

### 4.2 AD and Delusion

Three studies exhibit structural deviations in frontal and temporal lobes in AD patients with delusion (Qian, Schweizer, et al. 2019; Bruen et al. 2008; Nakaaki et al. 2013). The first one was a longitudinal study in 2012 on 53 AD patients (mean age: 78, female: 73.5%, MMSE score: 21.1, AD confirmation: NINCDS/ADRDA) of whom 66% were AD patients with psychosis. This study revealed a potential association between delusional symptoms in AD patients with structural deviations in the frontal and MTLs (Nakaaki et al. 2013). The other study in 2018 was a cohort study on 59 patients with AD (mean age: 78, female: 80%, MMSE score: 18.0). Overall, 40% of patients with AD suffered from delusion. Neuroimaging in AD‐developed delusions demonstrated a decreased volume of gray matter in the frontal region but both groups revealed a decrease in gray matter within frontotemporal brain regions over time (Qian, Schweizer, et al. 2019). Another study in 2008 on 31 patients with AD (mean age: 77, female: 38.7%, MMSE score: 23.3, AD confirmation: NINCDS‐ADRDA) revealed a significant correlation between delusion severity and declined gray matter density in the right inferior parietal lobule (IPL) and inferior frontal gyrus as well as left claustrum and medial and inferior frontal gyri (Bruen et al. 2008). Moreover, a study by Manca et al. (2023) found that AD patients who develop delusion had greater GM volume loss in both the left and the right caudate nuclei. Moreover, longitudinal analysis showed greater GM loss in delusional patients in the bilateral medio‐temporal ROIs (bilateral parahippocampal gyri and left hippocampus), in the right anterior cingulate cortex, and in posterior hubs of the DMN (bilateral precuneus and left posterior cingulate cortex). Furthermore, another study demonstrated higher WMH volume in the left occipital lobe in AD patients with delusion (Fan et al. 2023).

Although the above three studies showed structural changes in the frontal and temporal lobes of the brain in AD patients with delusion compared to patients without delusion, a study in 1995 showed that structural changes in the brain in Alzheimer's patients with and without delusion are not different. This study was a cross‐sectional study on 32 patients with AD (mean age: 78, female: 65.6%, AD confirmation: DSM‐III‐R and CERAD) (Howanitz et al. 1995).

Some studies investigated the structural changes of the brain in Alzheimer's patients according to the type of delusion. A longitudinal study in 2017 investigated the structural changes of the brain of specific psychosis symptoms (nonpsychotic, paranoid, misidentification, mixed) in AD patients. Psychotic symptoms revealed a decrease in left parahippocampal gyrus volume. A significant reduction in left parahippocampal volume was seen in patients with the mixes and misidentification subtypes in comparison with the paranoid and nonpsychotic groups (McLachlan et al. 2018). A cross‐sectional study in 2011 on 113 AD patients (mean age: 75, female: 65.4%, MMSE score: 19.3, AD confirmation: DSM‐IV and NINCDS/ADRDA) revealed a reduction in cortical thickness within the left superior temporal and left medial orbitofrontal regions in female patients with paranoid delusion compared to male patients with delusion (Whitehead et al. 2012).

A cohort study in 2019 using fMRI on 30 patients with AD (mean age: 76, female: 63.3%, AD confirmation: NIA‐AA) demonstrated a significant reduction in connectivity between the left IPL and the remaining regions within the DMN in AD patients with delusion (Qian, Fischer, et al. 2019).

### 4.3 AD and Hallucination

A cross‐sectional study in 1995 revealed an association between hallucination symptoms and overall brain atrophy, right and left lateral ventricle size (Howanitz et al. 1995). Patients with AD who experienced visual hallucinations showed a significantly decreased occipital/whole brain ratio compared to AD patients without visual hallucinations (Holroyd et al. 2000). Another study demonstrated that AD patients with visual hallucinations had a higher occipital periventricular hyperintensities score in comparison with those without visual hallucinations (Lin et al. 2006).

#### 4.3.1 Computed Tomography (CT) Scan

AD patients with delusion demonstrated temporal horns larger to the right and the frontal horn to the left on CT scan in a cross‐sectional study (mean age: 76, MMSE score: 22, AD confirmation: NINCDS‐ADRDA) (Geroldi et al. 2002). A longitudinal study in 2011 on 259 AD patients (mean age: 75, female: 67.9%, MMSE score: 21.4, AD confirmation: NINCDS/ADRDA) revealed that delusion and hallucination were significantly associated with changes in left lacunes and basal ganglia (Palmqvist et al. 2011).

## 5 SPECT

### 5.1 AD and Psychosis

SPECT analysis in AD patients revealed declined perfusion levels in the right inferior temporal regions and inferolateral prefrontal cortex in female AD patients with psychotic symptoms in comparison with AD female patients with no psychotic symptoms. Conversely, higher perfusion levels in the right striatum were exhibited in male AD patients with psychosis in comparison with AD male patients with no psychotic symptoms (Moran et al. 2008).

### 5.2 AD and Delusion

Most of the studies show hypoperfusion in different regions of the right hemisphere in Alzheimer's patients with delusion symptoms. We will continue to review these studies. A cross‐sectional study of 33 patients with AD revealed a correlation between delusion symptoms and hypoperfusion in the right anterior hemisphere but no distinct focal regions were indicated (Staff et al. 1999). Another study also exhibited significant hypoperfusion within different brain regions of the right hemisphere, including the anterior cingulate gyri, parietal cortex, prefrontal cortex, and inferior to middle temporal cortex (Nakano et al. 2006). A cross‐sectional study also revealed hypoperfusion in temporal regions. In this study, there was a significant hypoperfusion in the bilateral temporal lobes, with a dominance toward the right hemisphere. Specifically, hypoperfusion was exhibited in the right amygdala and right inferior temporal gyrus, in comparison with AD patients without delusion (Nakatsuka et al. 2013). AD individuals with delusions of theft exhibited significantly reduced cerebral blood flow in the right media posterior parietal region in comparison with patients without delusions (Fukuhara et al. 2001). In a cross‐sectional study, AD patients with delusion revealed a significant hypoperfusion in the right anterior insula (Matsuoka et al. 2010). A group of AD patients with autobiographic delusions revealed a notable region of hypoperfusion in the right frontal lobe compared to AD patients without delusions and AD patients with a range of delusions but no autobiographic content. This region of hypoperfusion covers specific parts of Brodmann's areas 9 and 10 (Staff et al. 2000). In a cross‐sectional study, eight types of delusions were classified by factor analysis and evaluated with a neuropsychiatric inventory. Delusion beliefs about home, phantom border symptom, abandonment, and misidentification were correlated with perfusion changes in the right temporal pole, medial frontal, and precentral regions. Delusion regarding television and persecution, with corresponding perfusion changes in the precuneus, insula, and thalamus. Delusions of abandonment and jealousy are related to perfusion changes in the right inferior temporal and frontal regions, middle frontal gyrus, insula, and posterior cingulate gyrus (Nomura et al. 2012).

Although most studies indicated involvement in the right hemisphere in Alzheimer's patients with delusion, in some studies, hypoperfusion was observed in the left hemisphere. In a study, AD patients with delusions revealed hypoperfusion in the left frontal lobe relative to the right frontal lobe (26) (Table 3).

**TABLE 3:** Summary of laterality in neuroimaging findings.

Study	Type of psychosis	Imaging method	Affected brain region(s)	Laterality
Kotrla 1995	Delusions, Hallucinations	SPECT	Frontal lobe, parietal lobe	Left (delusions), right (hallucinations)
Hirono 1998	Delusions	PET, MRI	Inferior temporal gyrus, medial occipital region	Left
Staff 1999	Delusions	SPECT	Anterior hemisphere	Right
Staff 2000	Autobiographic delusions	SPECT	Frontal lobe (Brodmann areas 9 and 10)	Right
Holroyd 2000	Visual hallucinations	MRI	Occipital lobe	Left
Fukahara 2001	Delusion of theft	SPECT	Media posterior parietal region	Right
Geroldi 2002	Delusions	CT	Temporal horn, frontal horn	Right (temporal), left (frontal)
L. Sultzer 2003	Delusions	FDG‐PET	Prefrontal cortex (Brodmann areas 8, 10)	Right
Nakano 2006	Delusions	SPECT	Prefrontal cortex, anterior cingulate gyri, temporal cortices, parietal cortex	Right
Moran 2008	Psychosis	SPECT	Prefrontal cortex, temporal regions, striatum	Right (females), right (males)
Buren 2008	Psychosis	MRI	Inferior frontal gyrus, inferior parietal lobule, frontal gyri, claustrum	Bilateral (right dominant)
Matsuoka 2010	Delusions	SPECT	Anterior insula	Right
Whitehead 2012	Paranoid delusions	MRI	Medial orbitofrontal, superior temporal regions	Left
Nomura 2012	Delusions	SPECT	Temporal pole, medial frontal, precentral regions, precuneus, insula, thalamus, posterior cingulate gyrus	Right
Nakatsuka 2013	Delusions	SPECT	Temporal lobes, amygdala	Bilateral (right dominant)
Sultzer 2014	Delusions	FDG‐PET	Frontal cortex, temporal cortex, orbitofrontal cortex	Bilateral (right dominant)
McLachlan 2018	Psychosis	MRI	Parahippocampal gyrus	Left
Lee 2019	Psychosis	MRI	Hippocampus	Right
Qian 2019	Delusions	fMRI	Inferior parietal lobule	Left
D'Antonio 2019	Psychosis	MRI	Inferior temporal lobe, fusiform gyrus, insula	Right
Manca 2023	Delusions	MRI	Caudate nuclei, parahippocampal gyri, hippocampus, anterior cingulate cortex, precuneus, posterior cingulate cortex	Bilateral (right and left specific regions)

Abbreviations: AD, Alzheimer's disease; CBF, cerebral blood flow; CT, computed tomography; DMN, default mode network; DTI, diffusion tensor imaging; EEG, electroencephalogram; FDG‐PET, fluorodeoxyglucose positron emission tomography; fMRI, functional magnetic resonance imaging; GM, gray matter; IPL, inferior parietal lobule; MRI, magnetic resonance imaging; MRS, magnetic resonance spectroscopy; PET, positron emission tomography; ROIs, regions of interest.; SPECT, single‐photon emission computed tomography; WMH, white matter hyperintensities.

### 5.3 AD and Hallucination

Patients presenting with hallucinations revealed hypoperfusion in the parietal lobe (Kotrla et al. 1995). According to the type of hallucination, AD patients who exhibited symptoms of visual and auditory hallucination revealed an imbalanced dysfunction in the frontal lobes as well as associated subcortical and parietal structures (Mega et al. 2000).

#### 5.3.1 PET Imaging

#### 5.3.2 AD and Psychosis

PET imaging analysis on AD patients with psychosis exhibited increases in tau pathology in occipital, frontal, and medial temporal cortexes compared to AD patients without psychotic symptoms (a cross‐sectional study on 67 AD patients, mean age: 79.2, female: 40.2%, MMSE score: 20.9, AD confirmation: NIA‐AA) (Gomar et al. 2022).

#### 5.3.3 AD and Delusion

Studies have shown that in Alzheimer's patients with delusion, there is dysfunction in different portions of the frontal and prefrontal cortex. In a cross‐sectional study using FDG‐PET, delusion was observed in AD patients who had dysfunction within specific regions of the frontal and temporal cortexes compared to AD patients without delusions. Patients with delusion revealed reduced metabolic activity in the right orbitofrontal cortex, lateral frontal cortex, and bilateral temporal cortex (Sultzer et al. 2014). Another study using FDG‐PET imaging revealed that the manifestation of delusion among AD patients was correlated with hypometabolism in two distinct regions of the right prefrontal cortex, including the inferior frontal pole (Brodmann's area 10) and the superior dorsolateral area (specifically the lateral aspect of Brodmann's area 8) (Sultzer et al. 2003). In this study, they did not compare results in AD patients with delusion symptoms to AD patients without delusion symptoms. The result of this study was in accordance with the result of another study using SPECT imaging that revealed hypoperfusion in specific parts of Brodmann's areas 9 and 10 in AD patients with delusion (Staff et al. 2000). Moreover, in a cross‐sectional study, AD patients with delusion symptoms had a significant increase in the metabolism of glucose in the left inferior temporal gyrus, whereas glucose metabolism was reduced in the left medial occipital region in comparison with those without delusions (Hirono et al. 1998).

### 5.4 Electroencephalogram (EEG) and magnetic resonance spectroscopy (MRS)

A cohort study on 173 AD patients (mean age: 69.4, female: 10.9%, MMSE score: 24, AD confirmation: NINCDS‐ADRDA) revealed AD patients with hallucination had lower peak frequency, a2‐functional connectivity, and a2‐ and b‐power but higher d‐power compared to AD patients without hallucination (Dauwan et al. 2018). Another study showed that AD patients with hallucinations had slightly higher ventricle‐brain ratios on CT scans and higher mean theta and delta powers on EEG (Förstl et al. 1993).

A cross‐sectional study using MRS in AD patients with psychosis demonstrated a significant reduction in *N*‐acetyl‐l‐aspartate and significant increases in glycerophosphoethanolamine. These results suggest that the excessive deterioration of synaptic integrity and neocortical neuronal may be an infrastructure for psychosis in AD patients (Sweet et al. 2002).

## 6 Discussion

**FIGURE 2:**
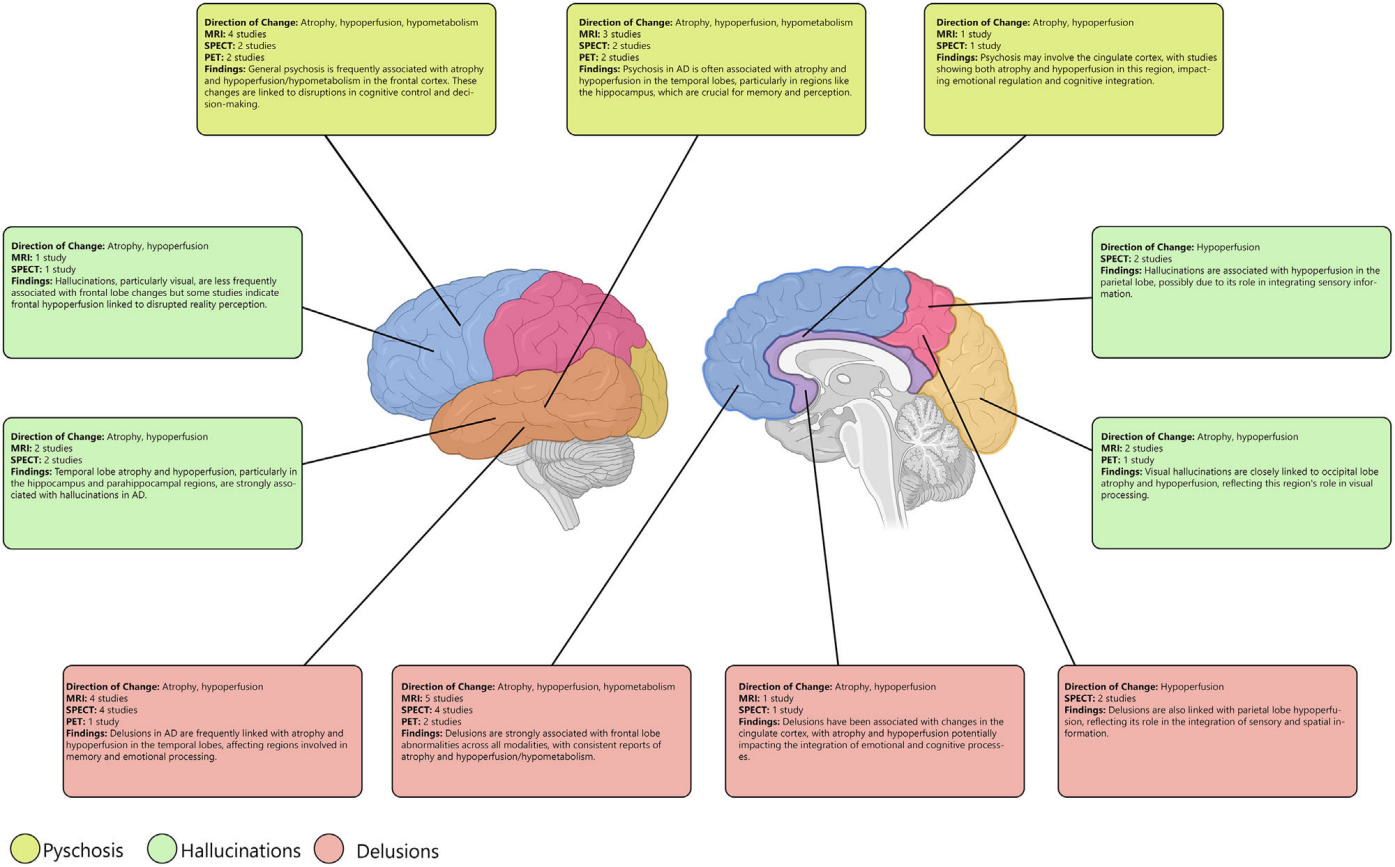
Summary of neuroimaging findings in Alzheimer's disease with delusion, hallucination, or psychosis.

The current review attempted to investigate neuroimaging findings of AD patients suffering from psychosis, hallucinations, and delusions. We reviewed studies using several imaging methods, including MRI, CT scan, SPECT, PET, MRS, and EEG. On the basis of the previous studies, there are significant changes in the volume and perfusion levels of broad brain areas, including the hippocampus, amygdala, insula, cingulate, occipital, frontal, prefrontal, orbitofrontal, temporal, and parietal cortices in these patients (Figure 2). Moreover, AD patients with psychosis, hallucinations, or delusions reflected different EEG waves compared to AD patients without these disorders.

The accumulation of Aβ plaques and synapse loss are the hallmark underlying mechanisms of AD (O'Brien and Wong 2011; Hong et al. 2016). Recent studies reported plenty of neuropathological mechanisms for psychosis in AD patients which mostly seem to be more severe than patients with AD only. Reduced amounts of Aβ1‐40 were reported in AD patients suffering from psychosis, revealing an increased Aβ1‐42/Aβ1‐40 ratio in the prefrontal cortex of these patients (Murray et al. 2012; Zhao et al. 2024). Moreover, increased neurofibrillary tangle (NFT) area density was reported in the neocortex region of patients with AD and psychosis (Farber et al. 2000). Loss of synaptic functions was considered the most important underlying factor in AD patients which seems to be more severe in AD patients with psychosis problems (Murray, Kumar, et al. 2014; Hao et al. 2023). These patients showed higher nucleus accumbens dopamine D3 receptor density but lower serotonin (5‐HT) in the ventral temporal cortex compared to patients with AD only (Sweet et al. 2001; Garcia‐Alloza et al. 2005; Marcos et al. 2008).

The MTL is a part of the brain that plays an important role in episodic memory (Karnik‐Henry et al. 2012). This region is shown to be the first brain area affected in AD reflecting volume atrophy and NFT accumulation (Braak and Braak 1991; Pini et al., Sep 2016; Hao et al. 2023). MTL consists of several subregions such as the hippocampus which makes two general networks, including anterior‐temporal (AT) and posterior‐medial (PM) networks (Ranganath and Ritchey 2012). Flores and colleagues reported different patterns for the alterations of AT and PM in AD patients. The results of their study showed higher tau uptake but lower amyloid uptake in the AT network compared to the PM network (de Flores et al. 2022; Zhang et al. 2021). Moreover, it is shown that decreased functional connectivity in the temporal regions can explain auditory hallucinations as well as psychosis (Hwang et al. 2021). Our study suggested MTL alterations, including atrophy and hypoperfusion in AD patients with psychosis or delusion by using various imaging methods.

The frontal lobe, as an essential part of the human brain, is involved in various cognitive processes, including long‐term planning, short‐term memory, and self‐reflection (Fuster 2001; Miller 2000; Fleming and Dolan 2012). Some studies suggested that white matter alterations in AD patients can result from vascular impairments such as ischemia in the frontal lobe (Englund et al. 1988). Farber et al. (2000), in their study, reported a more effective role for NFTs in the frontal area of these patients. It is shown that female psychotic AD patients, unlike male patients, may present more rapid tau accumulation in the frontal lobe compared to nonpsychotic AD patients (Koppel et al. 2014; Luo et al. 2019). This study also revealed an α‐synuclein‐related pathology in the frontal lobe of male psychotic AD patients (Koppel et al. 2014). Previous studies demonstrated that α‐synuclein pathology is significantly correlated with delusions and dementia with Lewy bodies (DLB) (McKeith 2006). Incorporating Lewy Body (LB) co‐pathology into the understanding of psychosis in AD is crucial, as it significantly influences both the clinical presentation and neuroimaging findings (Gibson et al. 2023; Liu et al. 2024). LB pathology, often present in AD, exacerbates neuropsychiatric symptoms, particularly hallucinations and delusions, by contributing to more pronounced atrophy in frontal and temporal regions (Devanand et al. 2022). This co‐pathology may also explain some inconsistencies in imaging studies, as patients with LB co‐pathology exhibit distinct atrophy patterns compared to those with pure AD (Chung and Kim 2015). Recognizing LB co‐pathology's role is essential for accurate interpretation of neuroimaging results and for tailoring clinical interventions. The results of our study showed the alteration in the frontal lobe as one of the most common findings in AD patients with psychosis. Moreover, structural alterations and dysfunction of the frontal lobe were reflected in AD patients with delusion and hallucination.

A notable observation is that many studies report findings predominantly in the right hemisphere. This includes regions such as the right prefrontal cortex, right inferior temporal gyrus, and right parietal lobe. This may suggest a lateralized vulnerability to psychosis‐related symptoms in AD, particularly in the right hemisphere. Although less frequent, there are significant findings in the left hemisphere, especially in cases involving delusions with reductions in glucose metabolism or structural changes in the left parahippocampal gyrus, frontal lobe, and occipital regions.

It is shown that the prefrontal lobe is another brain area that has an important impact on cognitive processes (Miller 2000; Duan et al. 2024). Dolotov et al. (2022) reported dysfunction of astrocytes and atrophic alterations in the prefrontal lobe as vital underlying mechanisms in both AD and depression disorders. Moreover, previous studies revealed a significant correlation between the atrophy of the prefrontal lobe as well as the MTL and dementia, which can be a strong predictor in patients with AD or depression (Burgmans et al. 2009). Moreover, reduced prefrontal thickness is reported as an indicator of negative symptom progression and cognitive impairments in patients with early psychosis (Tronchin et al. 2020; Wang, Wang, et al. 2022). Some studies suggested that there is an increased amount of phosphorylated tau in the dorsolateral prefrontal cortex (DLPFC) in AD patients with psychosis compared to AD patients without psychosis (Murray, Kirkwood, et al. 2014). Similar results were reported for NFT accumulation in this area of psychotic AD patients (Farber et al. 2000). Our study indicated dysfunction and decreased perfusion levels in the prefrontal region of AD patients with psychosis and delusion using SPECT and PET imaging methods.

The parietal lobe, especially the left parietal, is shown to be engaged in social cognition and language tasks (McKeith 2006; Li et al. 2022). Recent studies revealed a significant decrease in the metabolism of the parietal lobe of AD patients (Ma et al. 2018). This hypometabolism seems to be more severe in the medial parietal regions of AD patients (Imabayashi et al. 2004; Villain et al. 2010). Moreover, some studies reported reduced volume of the inferior parietal lobe in the early stages of AD (McDonald et al. 2009). Borgwardt et al. (2008), in a longitudinal study, showed a significant reduction in the gray matter volume of the parietal lobe, particularly the medial and superior parietal lobe, in patients with psychosis. Moreover, a significant correlation was shown between impairments of functional connectivity in the parietal memory network (PMN) and auditory hallucinations in patients with schizophrenia (Guo et al. 2020). Our study suggested hypoperfusion and dysfunction of different parietal areas as an underlying mechanism in AD patients with delusion and hallucination.

The most consistent finding across studies using MRI is the association between psychosis (particularly delusions and hallucinations) and structural abnormalities in the frontal, temporal, and parietal lobes. Specifically, atrophy or decreased gray matter volume in regions such as the inferior frontal gyrus, medial temporal regions, and parietal lobes has been frequently reported (Rao and Lyketsos 1998). Moreover, several studies point to a reduction in hippocampal and parahippocampal volumes, particularly in the right hemisphere, as being linked to the emergence of psychotic symptoms. These findings suggest that the neurodegenerative process in AD particularly affects regions involved in cognitive control, memory processing, and perception, which could contribute to the development of psychosis (Thompson et al. 2001; Gan et al. 2024). Consistent with MRI findings, SPECT studies reveal a pattern of reduced cerebral perfusion in regions corresponding to the frontal, temporal, and parietal cortices in AD patients with psychosis. Particularly, hypoperfusion in the right frontal lobe and the temporal‐parietal junction appears to be a recurrent finding. These areas are implicated in executive function and sensory integration, which might explain the occurrence of delusions and hallucinations in this patient population (McKhann et al. 2011).

This review included some limitations that are worth mentioning. First, most included studies lack longitudinal evaluation to provide sufficient evidence over time. In addition, there was no assessment of sex differences in AD patients with psychosis, hallucinations, or delusions to show various alterations between the two genders. Moreover, there were no adjustments for medications used by patients with psychosis symptoms. There was a lack of DTI and functional MRI studies to capture more information on the changes in tracts and connections responsible for psychosis, hallucinations, or delusions in AD patients. There was notable variability across studies regarding the specific regions implicated and the extent of the changes observed. Methodological differences, such as variations in imaging techniques, the severity of AD among patient populations, and differences in how psychosis is defined and measured, likely contribute to these inconsistencies. The heterogeneity of psychosis symptoms in AD, ranging from delusions to hallucinations with different underlying mechanisms, might also explain why some studies emphasize different brain regions. For instance, hallucinations are often linked with occipital lobe abnormalities, which are less consistently reported in studies focused on delusions. Sample size and demographic factors, such as age, sex, and the presence of comorbidities, might also account for the variability in findings. Smaller studies might be underpowered to detect certain effects, whereas differences in patient characteristics can introduce variability.

In conclusion, although there is consistent evidence that frontal and temporal lobe abnormalities are associated with psychosis in AD, the exact patterns of these abnormalities can vary depending on the symptom profile and the imaging modality used. The findings suggest that psychosis in AD may result from a complex interplay of structural, functional, and neurochemical alterations primarily involving the frontal‐temporal network. The results of our review provided evidence about the neuroimaging alterations in AD patients suffering from psychosis, hallucinations, and delusions using different imaging methods. AD patients with psychosis, hallucinations, or delusions have significant differences in the volume and perfusion levels of various brain regions along with alterations in EEG waves and biological molecules compared to patients with only AD. Further studies with larger sample sizes are needed to investigate tracts and connections affected in AD patients with psychosis, hallucinations, or delusions.

## Author Contributions

**Fardin Nabizadeh**: conceptualization, investigation, writing–original draft, visualization, methodology, writing–review and editing, validation, software, formal analysis, project administration, resources, supervision, data curation. **Shadi Sheykhlou**: data curation, resources, investigation. **Sara Mahmoodi**: investigation, data curation. **Elham Khalili**: writing–original draft, writing–review and editing, formal analysis. **Rasa Zafari**: formal analysis, writing–review and editing, writing–original draft. **Helia Hosseini**: writing–review and editing, writing–original draft.

## Ethics Statement

This article is based on previously conducted studies and does not contain any new studies with human participants or animals performed by any of the authors.

## Consent

This manuscript has been approved for publication by all authors.

## Conflicts of Interest

The authors declare no conflicts of interest.

### Peer Review

The peer review history for this article is available at https://publons.com/publon/10.1002/brb3.70205

## Supporting information



Supporting Information 1. Search strategy.

Supporting Information 2. Results of quality assessments.

## Data Availability

The datasets analyzed during the current study are available upon request with no restriction.
