# Supplementary material for: Neuroimaging Findings of Psychosis in Alzheimer's Disease: A Systematic Review
Source: Brain Behav. 2024 Dec 31;15(1):e70205. doi: 10.1002/brb3.70205 (PMC11688121; doi:10.1002/brb3.70205)
Supplement: Supplementary file 1 — Supporting Information 1. Search strategy. [file BRB3-15-e70205-s002.docx]

**Supplementary 1.** Search strategy

PubMed:387

(Alzheimer*[Title]) AND (psychosis OR hallucination OR charles bonnet syndrome OR delusion)

AND

(((positron emission tomography) OR PET or beta-amyloid or amyloid or amyloid-β or (amyloid deposition) OR PiB OR pittsburgh OR florbetapir OR flortaucipir OR tau OR (tau deposition) OR ‘FDG’ OR ‘fluorodeoxyglucose’)

OR

(DTI OR (diffusion tensor imaging) OR microstructure OR anisotropy OR Diffusivity)

OR

((functional magnetic resonance imaging) OR (functional MRI) OR fMRI OR rsfMRI OR (resting-state fMRI))

OR

((Brain mapping) OR (Structural MRI) OR (voxel-based morphometry) OR (gray matter) OR (white matter) OR VBM OR MRI OR (magnetic resonance imaging) OR atrophy OR hippocampus)

OR

(EEG OR electroencephalography OR Electroencephalogram)

OR

(SPECT OR (single-photon emission CT)))

Scopus: 530

((Alzheimer* OR Alzheimer’s disease) AND ((psychosis) OR (hallucination) OR (charles bonnet syndrome) OR (delusion)))

AND

(((positron emission tomography) OR PET or beta-amyloid or amyloid or amyloid-β or (amyloid deposition) OR PiB OR pittsburgh OR florbetapir OR flortaucipir OR tau OR (tau deposition) OR ‘FDG’ OR ‘fluorodeoxyglucose’)

OR

(DTI OR (diffusion tensor imaging) OR microstructure OR anisotropy OR Diffusivity)

OR

((functional magnetic resonance imaging) OR (functional MRI) OR fMRI OR rsfMRI OR (resting-state fMRI))

OR

((Brain mapping) OR (Structural MRI) OR (voxel-based morphometry) OR (gray matter) OR (white matter) OR VBM OR MRI OR (magnetic resonance imaging) OR atrophy OR hippocampus)

OR

(EEG OR electroencephalography OR Electroencephalogram)

OR

(SPECT OR (single-photon emission CT)))

WOS:87

(((“Alzheimer*)) AND (“psychosis)) OR “hallucination)) OR “charles bonnet syndrome)) OR delusion)))

AND

(TS=(positron emission tomography)) OR (TS=(PET)) OR (TS=(beta-amyloid)) OR (TS=(amyloid)) OR (TS=(amyloid-β)) OR (TS=(amyloid deposition)) OR (TS=(PiB)) OR (TS=(Pittsburgh)) OR (TS=(florbetapir)) OR (TS=(flortaucipir)) OR (TS=(tau)) OR (TS=(tau deposition)) OR (TS=(FDG)) OR (TS=(fluorodeoxyglucose)) OR (TS=(DTI)) OR (TS=(diffusion tensor imaging)) OR (TS=(microstructure)) OR (TS=(anisotropy)) OR (TS=(Diffusivity)) OR (TS=(functional magnetic resonance imaging)) OR (TS=(functional MRI)) OR (TS=(fMRI)) OR (TS=(rsfMRI)) OR (TS=(resting-state fMRI)) OR (TS=(Brain mapping)) OR (TS=(Structural MRI)) OR (TS=(voxel-based morphometry)) OR (TS=(gray matter)) OR (TS=(white matter)) OR (TS=(VBM)) OR (TS=(MRI)) OR (TS=(magnetic resonance imaging)) OR (TS=(atrophy)) OR (TS=(hippocampus)) OR (TS=(EEG)) OR (TS=(electroencephalography)) OR (TS=(Electroencephalogram))
