# Supplementary material for: Neuroimaging Findings of Psychosis in Alzheimer's Disease: A Systematic Review
Source: Brain Behav. 2024 Dec 31;15(1):e70205. doi: 10.1002/brb3.70205 (PMC11688121; doi:10.1002/brb3.70205)
Supplement: Supplementary file 2 — Supporting Information 2. Results of quality assessments. [file BRB3-15-e70205-s001.docx]

| Supplementary 2. Results of Quality assessments | | | | | |
| --- | --- | --- | --- | --- | --- |
| Author | Year | Selection | Comparability | Outcome, or Exposure | Total score |
| Fan | 2023 | 3 | 1 | 3 | 6 |
| Manca | 2023 | 4 | 1 | 2 | 7 |
| Gomar | 2022 | 3 | 1 | 2 | 6 |
| Lee | 2021 | 2 | 0 | 3 | 5 |
| Qian | 2019 | 3 | 1 | 2 | 6 |
| D'Antonio | 2019 | 4 | 1 | 3 | 8 |
| Lee | 2018 | 3 | 1 | 3 | 7 |
| Qian | 2018 | 3 | 1 | 2 | 6 |
| Dauwan | 2018 | 4 | 1 | 3 | 8 |
| McLachlan | 2017 | 3 | 1 | 2 | 6 |
| Makovac | 2015 | 4 | 1 | 3 | 8 |
| Nakatsuka | 2013 | 4 | 1 | 3 | 8 |
| L. Sultzer | 2013 | 3 | 1 | 3 | 7 |
| Nomura | 2012 | 3 | 1 | 3 | 7 |
| Nakaaki | 2012 | 4 | 1 | 3 | 8 |
| Palmqvist | 2011 | 4 | 1 | 3 | 8 |
| Whitehead | 2011 | 2 | 1 | 2 | 5 |
| Matsuoka | 2010 | 4 | 1 | 2 | 7 |
| Buren | 2008 | 4 | 1 | 3 | 8 |
| Moran | 2007 | 4 | 1 | 2 | 7 |
| Lin | 2006 | 4 | 1 | 3 | 8 |
| Nakano | 2006 | 3 | 1 | 2 | 6 |
| L. Sultzer | 2003 | 4 | 1 | 2 | 7 |
| Geroldi | 2002 | 4 | 1 | 3 | 8 |
| Sweet | 2001 | 4 | 1 | 2 | 7 |
| Fukahara | 2001 | 4 | 1 | 3 | 8 |
| Mega | 2000 | 4 | 1 | 3 | 8 |
| Staff | 2000 | 4 | 1 | 3 | 8 |
| Holroyd | 2000 | 4 | 1 | 2 | 7 |
| Staff | 1999 | 4 | 1 | 2 | 7 |
| Hirono | 1998 | 4 | 1 | 2 | 7 |
| Kotrla | 1995 | 4 | 1 | 3 | 8 |
| Howanitz | 1995 | 4 | 1 | 3 | 8 |
| Forstl | 1994 | 3 | 1 | 2 | 6 |
| NR: Not Reported | | | | | |
